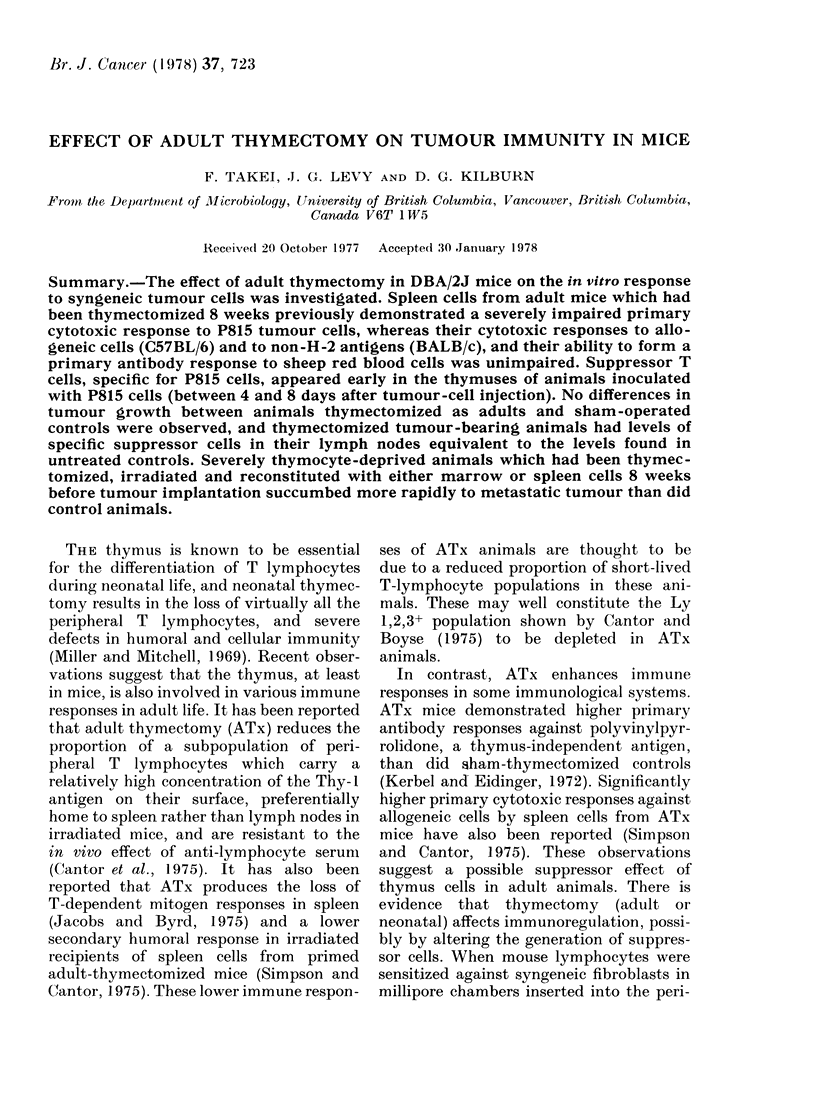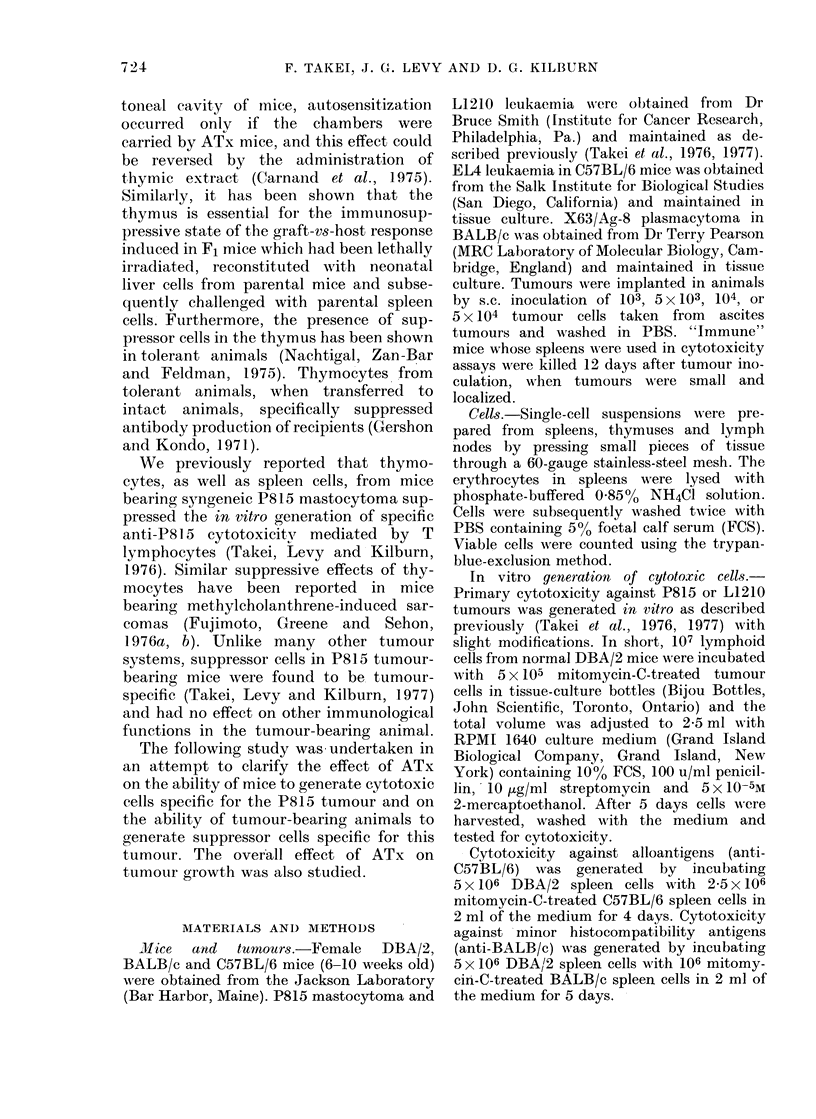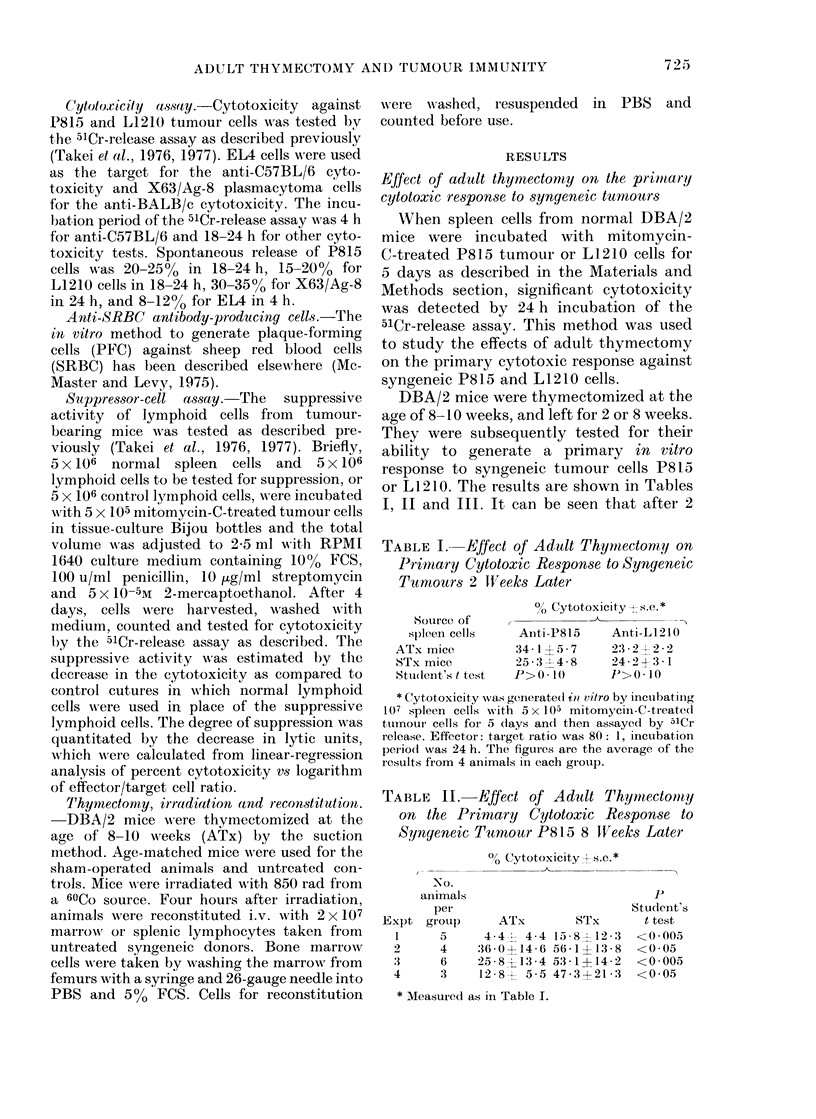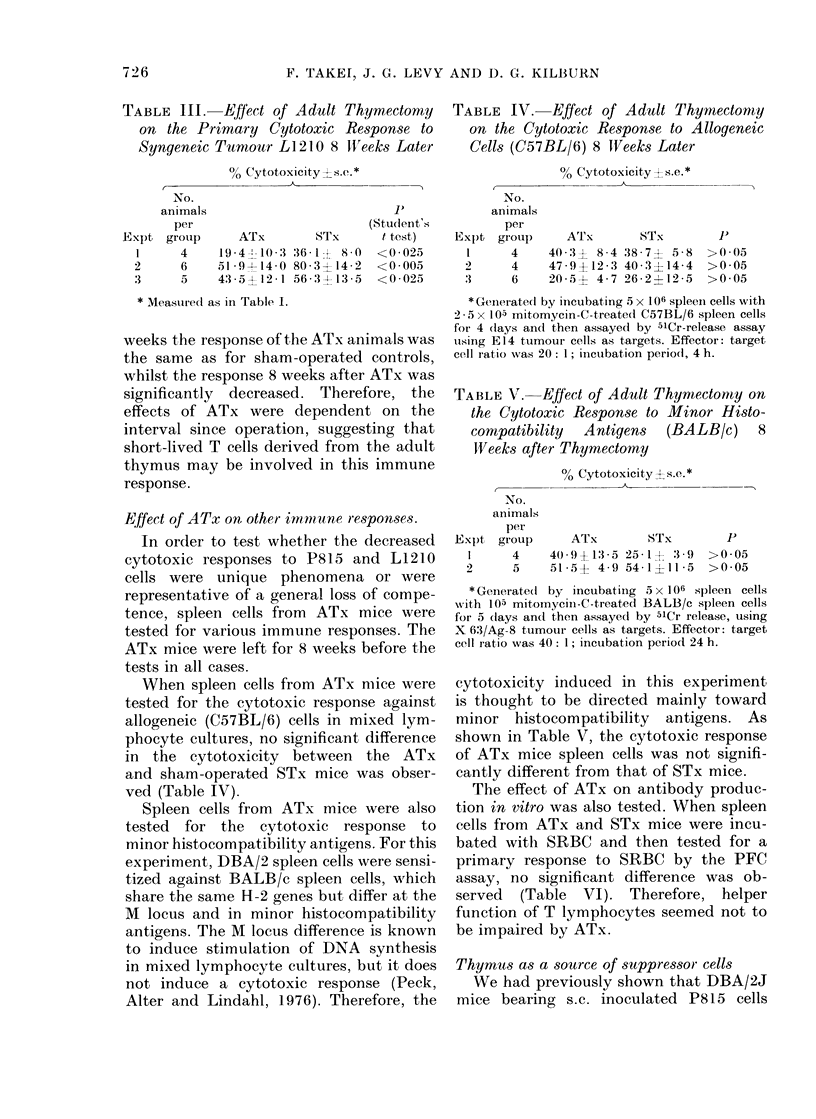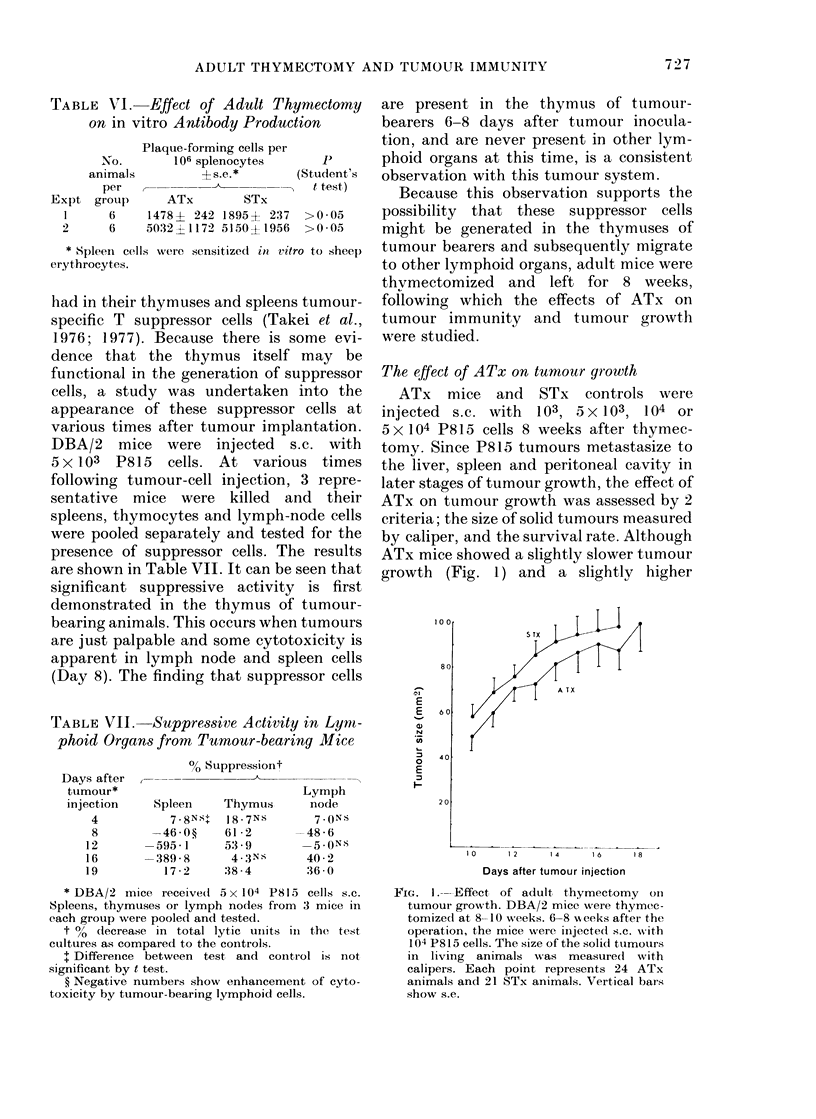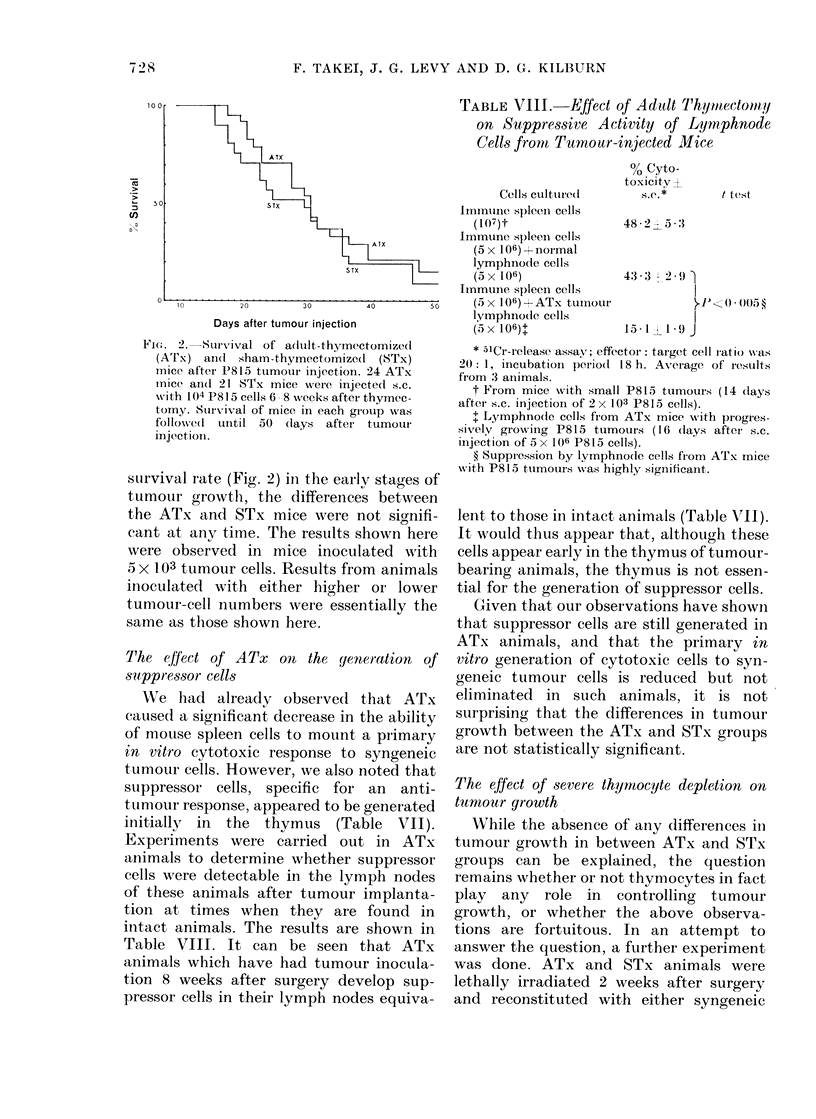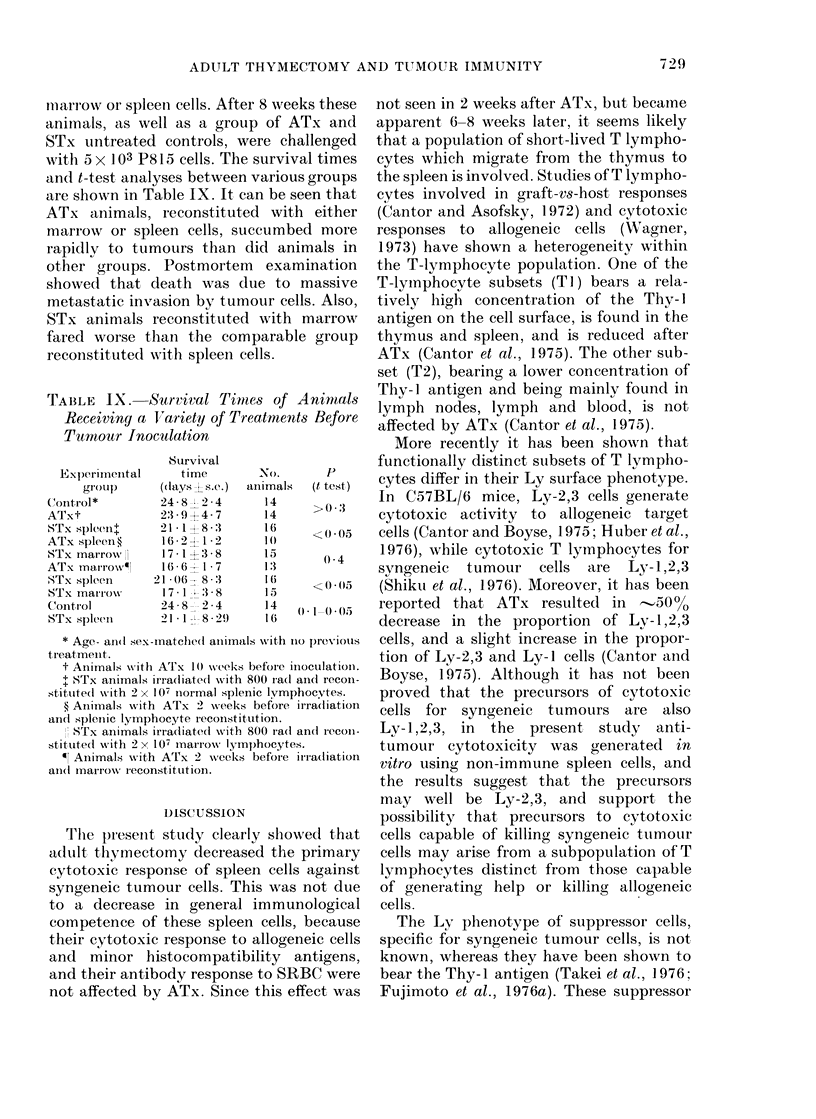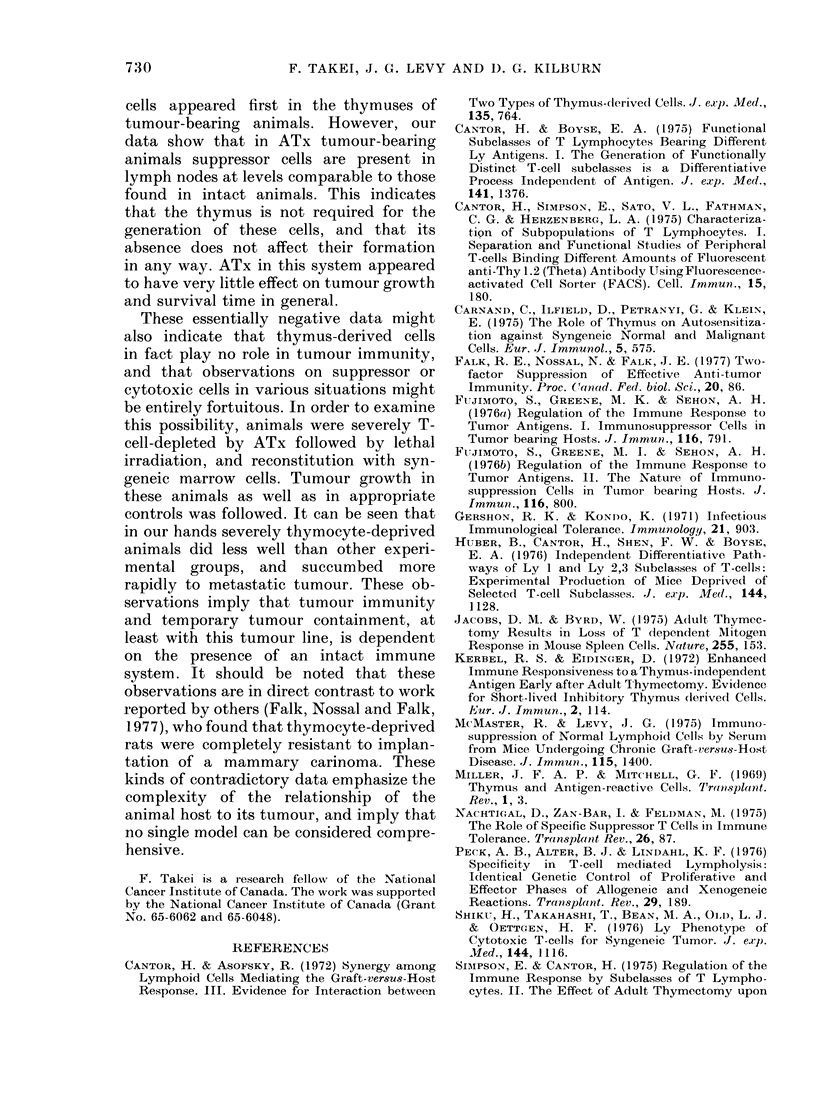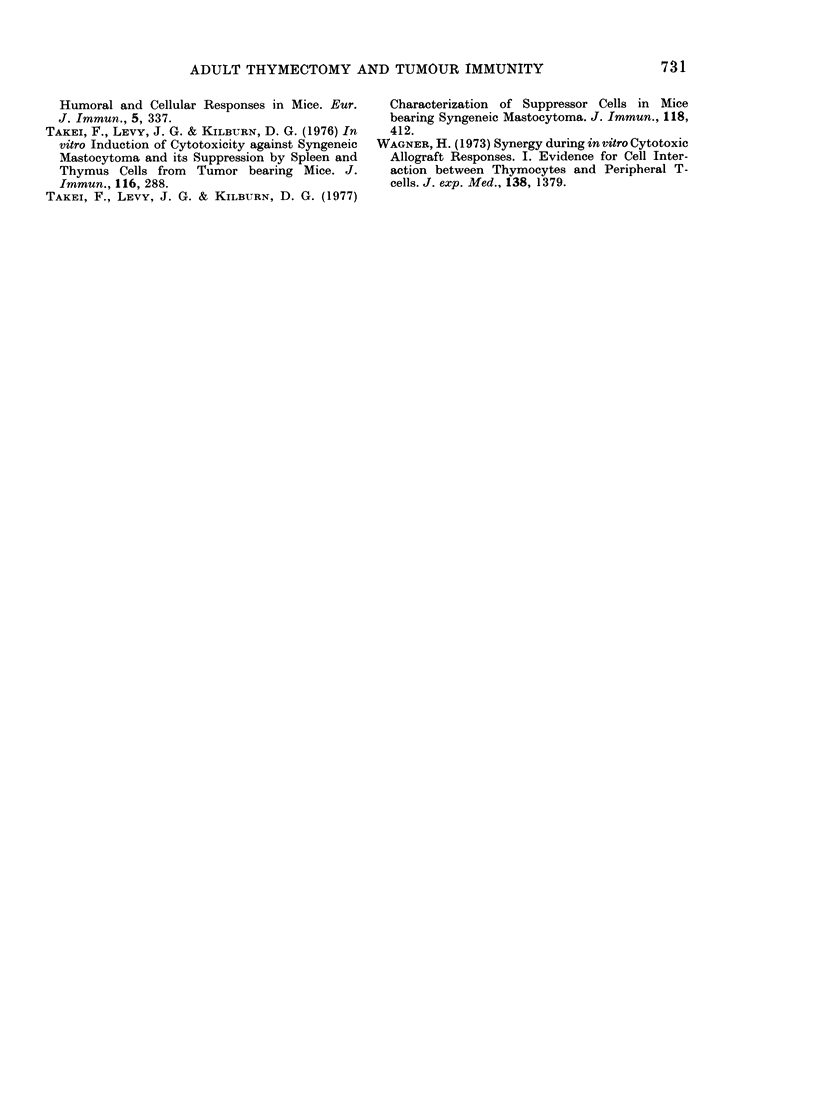# Effect of adult thymectomy on tumour immunity in mice.

**DOI:** 10.1038/bjc.1978.110

**Published:** 1978-05

**Authors:** F. Takei, J. G. Levy, D. G. Kilburn

## Abstract

The effect of adult thymectomy in DBA/2J mice on the in vitro response to syngeneic tumour cells was investigated. Spleen cells from adult mice which had been thymectomized 8 weeks previously demonstrated a severely impaired primary cytotoxic response to P815 tumour cells, whereas their cytotoxic responses to allogeneic cells (C57BL/6) and to non-H-2 antigens (BALB/c), and their ability to form a primary antibody response to sheep red blood cells was unimpaired. Suppressor T cells, specific for P815 cells, appeared early in the thymuses of animals inoculated with P815 cells (between 4 and 8 days after tumour-cell injection). No differences in tumour growth between animals thymectomized as adults and sham-operated controls were observed, and thymectomized tumour-bearing animals had levels of specific suppressor cells in their lymph nodes equivalent to the levels found in untreated controls. Severely thymocyte-deprived animals which had been thymectomized, irradiated and reconstituted with either marrow or spleen cells 8 weeks before tumour implantation succumbed more rapidly to metastatic tumour than did control animals.


*Br. J. Cancer* (1978) **37**, 723

### EFFECT OF ADULT THYMECTOMY ON TUMOUR IMMUNITY IN MICE

F. TAKEI, J. G. LEVY AND D. G. KILBURN

*From the Department of Microbiology, University of British Columbia, Vancouver, British Columbia, Canada V6T 1W5*

Received 20 October 1977 Accepted 30 January 1978

**Summary.—The effect of adult thymectomy in DBA/2J mice on the *in vitro* response to syngeneic tumour cells was investigated. Spleen cells from adult mice which had been thymectomized 8 weeks previously demonstrated a severely impaired primary cytotoxic response to P815 tumour cells, whereas their cytotoxic responses to allogeneic cells (C57BL/6) and to non-H-2 antigens (BALB/c), and their ability to form a primary antibody response to sheep red blood cells was unimpaired. Suppressor T cells, specific for P815 cells, appeared early in the thymuses of animals inoculated with P815 cells (between 4 and 8 days after tumour-cell injection). No differences in tumour growth between animals thymectomized as adults and sham-operated controls were observed, and thymectomized tumour-bearing animals had levels of specific suppressor cells in their lymph nodes equivalent to the levels found in untreated controls. Severely thymocyte-deprived animals which had been thymectomized, irradiated and reconstituted with either marrow or spleen cells 8 weeks before tumour implantation succumbed more rapidly to metastatic tumour than did control animals.**

THE thymus is known to be essential for the differentiation of T lymphocytes during neonatal life, and neonatal thymectomy results in the loss of virtually all the peripheral T lymphocytes, and severe defects in humoral and cellular immunity (Miller and Mitchell, 1969). Recent observations suggest that the thymus, at least in mice, is also involved in various immune responses in adult life. It has been reported that adult thymectomy (ATx) reduces the proportion of a subpopulation of peripheral T lymphocytes which carry a relatively high concentration of the Thy-1 antigen on their surface, preferentially home to spleen rather than lymph nodes in irradiated mice, and are resistant to the *in vivo* effect of anti-lymphocyte serum (Cantor *et al.*, 1975). It has also been reported that ATx produces the loss of T-dependent mitogen responses in spleen (Jacobs and Byrd, 1975) and a lower secondary humoral response in irradiated recipients of spleen cells from primed adult-thymectomized mice (Simpson and Cantor, 1975). These lower immune responses of ATx animals are thought to be due to a reduced proportion of short-lived T-lymphocyte populations in these animals. These may well constitute the Ly $1,2,3^+$ population shown by Cantor and Boyse (1975) to be depleted in ATx animals.

In contrast, ATx enhances immune responses in some immunological systems. ATx mice demonstrated higher primary antibody responses against polyvinylpyrrolidone, a thymus-independent antigen, than did sham-thymectomized controls (Kerbel and Eidinger, 1972). Significantly higher primary cytotoxic responses against allogeneic cells by spleen cells from ATx mice have also been reported (Simpson and Cantor, 1975). These observations suggest a possible suppressor effect of thymus cells in adult animals. There is evidence that thymectomy (adult or neonatal) affects immunoregulation, possibly by altering the generation of suppressor cells. When mouse lymphocytes were sensitized against syngeneic fibroblasts in millipore chambers inserted into the peritoneal cavity of mice, autosensitization occurred only if the chambers were carried by ATx mice, and this effect could be reversed by the administration of thymic extract (Carnand *et al.*, 1975). Similarly, it has been shown that the thymus is essential for the immunosuppressive state of the graft-*vs*-host response induced in $F_1$ mice which had been lethally irradiated, reconstituted with neonatal liver cells from parental mice and subsequently challenged with parental spleen cells. Furthermore, the presence of suppressor cells in the thymus has been shown in tolerant animals (Nachtigal, Zan-Bar and Feldman, 1975). Thymocytes from tolerant animals, when transferred to intact animals, specifically suppressed antibody production of recipients (Gershon and Kondo, 1971).

We previously reported that thymocytes, as well as spleen cells, from mice bearing syngeneic P815 mastocytoma suppressed the *in vitro* generation of specific anti-P815 cytotoxicity mediated by T lymphocytes (Takei, Levy and Kilburn, 1976). Similar suppressive effects of thymocytes have been reported in mice bearing methylcholanthrene-induced sarcomas (Fujimoto, Greene and Sehon, 1976*a*, *b*). Unlike many other tumour systems, suppressor cells in P815 tumour-bearing mice were found to be tumour-specific (Takei, Levy and Kilburn, 1977) and had no effect on other immunological functions in the tumour-bearing animal.

The following study was undertaken in an attempt to clarify the effect of ATx on the ability of mice to generate cytotoxic cells specific for the P815 tumour and on the ability of tumour-bearing animals to generate suppressor cells specific for this tumour. The overall effect of ATx on tumour growth was also studied.

### MATERIALS AND METHODS

*Mice and tumours.*—Female DBA/2, BALB/c and C57BL/6 mice (6–10 weeks old) were obtained from the Jackson Laboratory (Bar Harbor, Maine). P815 mastocytoma and L1210 leukaemia were obtained from Dr Bruce Smith (Institute for Cancer Research, Philadelphia, Pa.) and maintained as described previously (Takei *et al.*, 1976, 1977). EL4 leukaemia in C57BL/6 mice was obtained from the Salk Institute for Biological Studies (San Diego, California) and maintained in tissue culture. X63/Ag-8 plasmacytoma in BALB/c was obtained from Dr Terry Pearson (MRC Laboratory of Molecular Biology, Cambridge, England) and maintained in tissue culture. Tumours were implanted in animals by s.c. inoculation of $10^3$, $5\times10^3$, $10^4$, or $5\times10^4$ tumour cells taken from ascites tumours and washed in PBS. "Immune" mice whose spleens were used in cytotoxicity assays were killed 12 days after tumour inoculation, when tumours were small and localized.

*Cells.*—Single-cell suspensions were prepared from spleens, thymuses and lymph nodes by pressing small pieces of tissue through a 60-gauge stainless-steel mesh. The erythrocytes in spleens were lysed with phosphate-buffered 0·85% $NH_4Cl$ solution. Cells were subsequently washed twice with PBS containing 5% foetal calf serum (FCS). Viable cells were counted using the trypan-blue-exclusion method.

In vitro *generation of cytotoxic cells.*—Primary cytotoxicity against P815 or L1210 tumours was generated *in vitro* as described previously (Takei *et al.*, 1976, 1977) with slight modifications. In short, $10^7$ lymphoid cells from normal DBA/2 mice were incubated with $5\times10^5$ mitomycin-C-treated tumour cells in tissue-culture bottles (Bijou Bottles, John Scientific, Toronto, Ontario) and the total volume was adjusted to 2·5 ml with RPMI 1640 culture medium (Grand Island Biological Company, Grand Island, New York) containing 10% FCS, 100 u/ml penicillin, 10 $\mu$g/ml streptomycin and $5\times10^{-5}$M 2-mercaptoethanol. After 5 days cells were harvested, washed with the medium and tested for cytotoxicity.

Cytotoxicity against alloantigens (anti-C57BL/6) was generated by incubating $5\times10^6$ DBA/2 spleen cells with $2{\cdot}5\times10^6$ mitomycin-C-treated C57BL/6 spleen cells in 2 ml of the medium for 4 days. Cytotoxicity against minor histocompatibility antigens (anti-BALB/c) was generated by incubating $5\times10^6$ DBA/2 spleen cells with $10^6$ mitomycin-C-treated BALB/c spleen cells in 2 ml of the medium for 5 days.

*Cytotoxicity assay.*—Cytotoxicity against P815 and L1210 tumour cells was tested by the $^{51}$Cr-release assay as described previously (Takei *et al.*, 1976, 1977). EL4 cells were used as the target for the anti-C57BL/6 cytotoxicity and X63/Ag-8 plasmacytoma cells for the anti-BALB/c cytotoxicity. The incubation period of the $^{51}$Cr-release assay was 4 h for anti-C57BL/6 and 18–24 h for other cytotoxicity tests. Spontaneous release of P815 cells was 20–25% in 18–24 h, 15–20% for L1210 cells in 18–24 h, 30–35% for X63/Ag-8 in 24 h, and 8–12% for EL4 in 4 h.

*Anti-SRBC antibody-producing cells.*—The *in vitro* method to generate plaque-forming cells (PFC) against sheep red blood cells (SRBC) has been described elsewhere (McMaster and Levy, 1975).

*Suppressor-cell assay.*—The suppressive activity of lymphoid cells from tumour-bearing mice was tested as described previously (Takei *et al.*, 1976, 1977). Briefly, $5 \times 10^6$ normal spleen cells and $5 \times 10^6$ lymphoid cells to be tested for suppression, or $5 \times 10^6$ control lymphoid cells, were incubated with $5 \times 10^5$ mitomycin-C-treated tumour cells in tissue-culture Bijou bottles and the total volume was adjusted to 2·5 ml with RPMI 1640 culture medium containing 10% FCS, 100 u/ml penicillin, 10 μg/ml streptomycin and $5 \times 10^{-5}$M 2-mercaptoethanol. After 4 days, cells were harvested, washed with medium, counted and tested for cytotoxicity by the $^{51}$Cr-release assay as described. The suppressive activity was estimated by the decrease in the cytotoxicity as compared to control cutures in which normal lymphoid cells were used in place of the suppressive lymphoid cells. The degree of suppression was quantitated by the decrease in lytic units, which were calculated from linear-regression analysis of percent cytotoxicity *vs* logarithm of effector/target cell ratio.

*Thymectomy, irradiation and reconstitution.*—DBA/2 mice were thymectomized at the age of 8–10 weeks (ATx) by the suction method. Age-matched mice were used for the sham-operated animals and untreated controls. Mice were irradiated with 850 rad from a $^{60}$Co source. Four hours after irradiation, animals were reconstituted i.v. with $2 \times 10^7$ marrow or splenic lymphocytes taken from untreated syngeneic donors. Bone marrow cells were taken by washing the marrow from femurs with a syringe and 26-gauge needle into PBS and 5% FCS. Cells for reconstitution were washed, resuspended in PBS and counted before use.

### RESULTS

### *Effect of adult thymectomy on the primary cytotoxic response to syngeneic tumours*

When spleen cells from normal DBA/2 mice were incubated with mitomycin-C-treated P815 tumour or L1210 cells for 5 days as described in the Materials and Methods section, significant cytotoxicity was detected by 24 h incubation of the $^{51}$Cr-release assay. This method was used to study the effects of adult thymectomy on the primary cytotoxic response against syngeneic P815 and L1210 cells.

DBA/2 mice were thymectomized at the age of 8–10 weeks, and left for 2 or 8 weeks. They were subsequently tested for their ability to generate a primary *in vitro* response to syngeneic tumour cells P815 or L1210. The results are shown in Tables I, II and III. It can be seen that after 2

TABLE I.—*Effect of Adult Thymectomy on Primary Cytotoxic Response to Syngeneic Tumours 2 Weeks Later*

| Source of spleen cells | % Cytotoxicity ± s.e.* | |
|---|---|---|
| | Anti-P815 | Anti-L1210 |
| ATx mice | 34·1 ± 5·7 | 23·2 ± 2·2 |
| STx mice | 25·3 ± 4·8 | 24·2 ± 3·1 |
| Student's *t* test | $P > 0·10$ | $P > 0·10$ |

* Cytotoxicity was generated *in vitro* by incubating $10^7$ spleen cells with $5 \times 10^5$ mitomycin-C-treated tumour cells for 5 days and then assayed by $^{51}$Cr release. Effector: target ratio was 80 : 1, incubation period was 24 h. The figures are the average of the results from 4 animals in each group.

TABLE II.—*Effect of Adult Thymectomy on the Primary Cytotoxic Response to Syngeneic Tumour P815 8 Weeks Later*

| Expt | No. animals per group | % Cytotoxicity ± s.e.* ATx | STx | *P* Student's *t* test |
|---|---|---|---|---|
| 1 | 5 | 4·4 ± 4·4 | 15·8 ± 12·3 | <0·005 |
| 2 | 4 | 36·0 ± 14·6 | 56·1 ± 13·8 | <0·05 |
| 3 | 6 | 25·8 ± 13·4 | 53·1 ± 14·2 | <0·005 |
| 4 | 3 | 12·8 ± 5·5 | 47·3 ± 21·3 | <0·05 |

* Measured as in Table I.

TABLE III.—*Effect of Adult Thymectomy on the Primary Cytotoxic Response to Syngeneic Tumour L1210 8 Weeks Later*

| Expt | No. animals per group | % Cytotoxicity ± s.e.* ATx | STx | P (Student's *t* test) |
|---|---|---|---|---|
| 1 | 4 | 19·4 ± 10·3 | 36·1 ± 8·0 | <0·025 |
| 2 | 6 | 51·9 ± 14·0 | 80·3 ± 14·2 | <0·005 |
| 3 | 5 | 43·5 ± 12·1 | 56·3 ± 13·5 | <0·025 |

* Measured as in Table I.

weeks the response of the ATx animals was the same as for sham-operated controls, whilst the response 8 weeks after ATx was significantly decreased. Therefore, the effects of ATx were dependent on the interval since operation, suggesting that short-lived T cells derived from the adult thymus may be involved in this immune response.

*Effect of ATx on other immune responses.*

In order to test whether the decreased cytotoxic responses to P815 and L1210 cells were unique phenomena or were representative of a general loss of competence, spleen cells from ATx mice were tested for various immune responses. The ATx mice were left for 8 weeks before the tests in all cases.

When spleen cells from ATx mice were tested for the cytotoxic response against allogeneic (C57BL/6) cells in mixed lymphocyte cultures, no significant difference in the cytotoxicity between the ATx and sham-operated STx mice was observed (Table IV).

Spleen cells from ATx mice were also tested for the cytotoxic response to minor histocompatibility antigens. For this experiment, DBA/2 spleen cells were sensitized against BALB/c spleen cells, which share the same H-2 genes but differ at the M locus and in minor histocompatibility antigens. The M locus difference is known to induce stimulation of DNA synthesis in mixed lymphocyte cultures, but it does not induce a cytotoxic response (Peck, Alter and Lindahl, 1976). Therefore, the

TABLE IV.—*Effect of Adult Thymectomy on the Cytotoxic Response to Allogeneic Cells (C57BL/6) 8 Weeks Later*

| Expt | No. animals per group | % Cytotoxicity ± s.e.* ATx | STx | P |
|---|---|---|---|---|
| 1 | 4 | 40·3 ± 8·4 | 38·7 ± 5·8 | >0·05 |
| 2 | 4 | 47·9 ± 12·3 | 40·3 ± 14·4 | >0·05 |
| 3 | 6 | 20·5 ± 4·7 | 26·2 ± 12·5 | >0·05 |

*Generated by incubating $5 \times 10^6$ spleen cells with $2·5 \times 10^5$ mitomycin-C-treated C57BL/6 spleen cells for 4 days and then assayed by $^{51}Cr$-release assay using E14 tumour cells as targets. Effector: target cell ratio was 20 : 1; incubation period, 4 h.

TABLE V.—*Effect of Adult Thymectomy on the Cytotoxic Response to Minor Histocompatibility Antigens (BALB/c) 8 Weeks after Thymectomy*

| Expt | No. animals per group | % Cytotoxicity ± s.e.* ATx | STx | P |
|---|---|---|---|---|
| 1 | 4 | 40·9 ± 13·5 | 25·1 ± 3·9 | >0·05 |
| 2 | 5 | 51·5 ± 4·9 | 54·1 ± 11·5 | >0·05 |

*Generated by incubating $5 \times 10^6$ spleen cells with $10^5$ mitomycin-C-treated BALB/c spleen cells for 5 days and then assayed by $^{51}Cr$ release, using X 63/Ag-8 tumour cells as targets. Effector: target cell ratio was 40 : 1; incubation period 24 h.

cytotoxicity induced in this experiment is thought to be directed mainly toward minor histocompatibility antigens. As shown in Table V, the cytotoxic response of ATx mice spleen cells was not significantly different from that of STx mice.

The effect of ATx on antibody production *in vitro* was also tested. When spleen cells from ATx and STx mice were incubated with SRBC and then tested for a primary response to SRBC by the PFC assay, no significant difference was observed (Table VI). Therefore, helper function of T lymphocytes seemed not to be impaired by ATx.

*Thymus as a source of suppressor cells*

We had previously shown that DBA/2J mice bearing s.c. inoculated P815 cells

TABLE VI.—*Effect of Adult Thymectomy on* in vitro *Antibody Production*

| Expt | No. animals per group | Plaque-forming cells per $10^6$ splenocytes ±s.e.* ATx | STx | P (Student's t test) |
|---|---|---|---|---|
| 1 | 6 | 1478± 242 | 1895± 237 | >0·05 |
| 2 | 6 | 5032±1172 | 5150±1956 | >0·05 |

* Spleen cells were sensitized *in vitro* to sheep erythrocytes.

had in their thymuses and spleens tumour-specific T suppressor cells (Takei *et al.*, 1976; 1977). Because there is some evidence that the thymus itself may be functional in the generation of suppressor cells, a study was undertaken into the appearance of these suppressor cells at various times after tumour implantation. DBA/2 mice were injected s.c. with $5\times10^3$ P815 cells. At various times following tumour-cell injection, 3 representative mice were killed and their spleens, thymocytes and lymph-node cells were pooled separately and tested for the presence of suppressor cells. The results are shown in Table VII. It can be seen that significant suppressive activity is first demonstrated in the thymus of tumour-bearing animals. This occurs when tumours are just palpable and some cytotoxicity is apparent in lymph node and spleen cells (Day 8). The finding that suppressor cells

TABLE VII.—*Suppressive Activity in Lymphoid Organs from Tumour-bearing Mice*

| Days after tumour* injection | % Suppression† Spleen | Thymus | Lymph node |
|---|---|---|---|
| 4 | 7·8NS‡ | 18·7NS | 7·0NS |
| 8 | −46·0§ | 61·2 | −48·6 |
| 12 | −595·1 | 53·9 | −5·0NS |
| 16 | −389·8 | 4·3NS | 40·2 |
| 19 | 17·2 | 38·4 | 36·0 |

* DBA/2 mice received $5\times10^4$ P815 cells s.c. Spleens, thymuses or lymph nodes from 3 mice in each group were pooled and tested.

† % decrease in total lytic units in the test cultures as compared to the controls.

‡ Difference between test and control is not significant by *t* test.

§ Negative numbers show enhancement of cytotoxicity by tumour-bearing lymphoid cells.

are present in the thymus of tumour-bearers 6–8 days after tumour inoculation, and are never present in other lymphoid organs at this time, is a consistent observation with this tumour system.

Because this observation supports the possibility that these suppressor cells might be generated in the thymuses of tumour bearers and subsequently migrate to other lymphoid organs, adult mice were thymectomized and left for 8 weeks, following which the effects of ATx on tumour immunity and tumour growth were studied.

*The effect of ATx on tumour growth*

ATx mice and STx controls were injected s.c. with $10^3$, $5\times10^3$, $10^4$ or $5\times10^4$ P815 cells 8 weeks after thymectomy. Since P815 tumours metastasize to the liver, spleen and peritoneal cavity in later stages of tumour growth, the effect of ATx on tumour growth was assessed by 2 criteria; the size of solid tumours measured by caliper, and the survival rate. Although ATx mice showed a slightly slower tumour growth (Fig. 1) and a slightly higher

FIG. 1.—Effect of adult thymectomy on tumour growth. DBA/2 mice were thymectomized at 8–10 weeks. 6–8 weeks after the operation, the mice were injected s.c. with $10^4$ P815 cells. The size of the solid tumours in living animals was measured with calipers. Each point represents 24 ATx animals and 21 STx animals. Vertical bars show s.e.

FIG. 2.—Survival of adult-thymectomized (ATx) and sham-thymectomized (STx) mice after P815 tumour injection. 24 ATx mice and 21 STx mice were injected s.c. with $10^4$ P815 cells 6–8 weeks after thymectomy. Survival of mice in each group was followed until 50 days after tumour injection.

TABLE VIII.—*Effect of Adult Thymectomy on Suppressive Activity of Lymphnode Cells from Tumour-injected Mice*

| Cells cultured | % Cytotoxicity ± s.e.* | t test |
|---|---|---|
| Immune spleen cells ($10^7$)† | 48·2 ± 5·3 | |
| Immune spleen cells ($5\times10^6$) + normal lymphnode cells ($5\times10^6$) | 43·3 ± 2·9 | $P<0\cdot005$§ |
| Immune spleen cells ($5\times10^6$) + ATx tumour lymphnode cells ($5\times10^6$)‡ | 15·1 ± 1·9 | |

* $^{51}$Cr-release assay; effector : target cell ratio was 20 : 1, incubation period 18 h. Average of results from 3 animals.

† From mice with small P815 tumours (14 days after s.c. injection of $2\times10^3$ P815 cells).

‡ Lymphnode cells from ATx mice with progressively growing P815 tumours (16 days after s.c. injection of $5\times10^6$ P815 cells).

§ Suppression by lymphnode cells from ATx mice with P815 tumours was highly significant.

survival rate (Fig. 2) in the early stages of tumour growth, the differences between the ATx and STx mice were not significant at any time. The results shown here were observed in mice inoculated with $5\times10^3$ tumour cells. Results from animals inoculated with either higher or lower tumour-cell numbers were essentially the same as those shown here.

*The effect of ATx on the generation of suppressor cells*

We had already observed that ATx caused a significant decrease in the ability of mouse spleen cells to mount a primary *in vitro* cytotoxic response to syngeneic tumour cells. However, we also noted that suppressor cells, specific for an anti-tumour response, appeared to be generated initially in the thymus (Table VII). Experiments were carried out in ATx animals to determine whether suppressor cells were detectable in the lymph nodes of these animals after tumour implantation at times when they are found in intact animals. The results are shown in Table VIII. It can be seen that ATx animals which have had tumour inoculation 8 weeks after surgery develop suppressor cells in their lymph nodes equivalent to those in intact animals (Table VII). It would thus appear that, although these cells appear early in the thymus of tumour-bearing animals, the thymus is not essential for the generation of suppressor cells.

Given that our observations have shown that suppressor cells are still generated in ATx animals, and that the primary *in vitro* generation of cytotoxic cells to syngeneic tumour cells is reduced but not eliminated in such animals, it is not surprising that the differences in tumour growth between the ATx and STx groups are not statistically significant.

*The effect of severe thymocyte depletion on tumour growth*

While the absence of any differences in tumour growth in between ATx and STx groups can be explained, the question remains whether or not thymocytes in fact play any role in controlling tumour growth, or whether the above observations are fortuitous. In an attempt to answer the question, a further experiment was done. ATx and STx animals were lethally irradiated 2 weeks after surgery and reconstituted with either syngeneic marrow or spleen cells. After 8 weeks these animals, as well as a group of ATx and STx untreated controls, were challenged with $5 \times 10^3$ P815 cells. The survival times and *t*-test analyses between various groups are shown in Table IX. It can be seen that ATx animals, reconstituted with either marrow or spleen cells, succumbed more rapidly to tumours than did animals in other groups. Postmortem examination showed that death was due to massive metastatic invasion by tumour cells. Also, STx animals reconstituted with marrow fared worse than the comparable group reconstituted with spleen cells.

TABLE IX.—*Survival Times of Animals Receiving a Variety of Treatments Before Tumour Inoculation*

| Experimental group | Survival time (days ± s.e.) | No. animals | P (*t* test) |
|---|---|---|---|
| Control* | 24·8 ± 2·4 | 14 | >0·3 |
| ATx† | 23·9 ± 4·7 | 14 | |
| STx spleen‡ | 21·1 ± 8·3 | 16 | <0·05 |
| ATx spleen§ | 16·2 ± 1·2 | 10 | |
| STx marrow‖ | 17·1 ± 3·8 | 15 | 0·4 |
| ATx marrow¶ | 16·6 ± 1·7 | 13 | |
| STx spleen | 21·06 ± 8·3 | 16 | <0·05 |
| STx marrow | 17·1 ± 3·8 | 15 | |
| Control | 24·8 ± 2·4 | 14 | 0·1–0·05 |
| STx spleen | 21·1 ± 8·29 | 16 | |

* Age- and sex-matched animals with no previous treatment.

† Animals with ATx 10 weeks before inoculation.

‡ STx animals irradiated with 800 rad and reconstituted with $2 \times 10^7$ normal splenic lymphocytes.

§ Animals with ATx 2 weeks before irradiation and splenic lymphocyte reconstitution.

‖ STx animals irradiated with 800 rad and reconstituted with $2 \times 10^7$ marrow lymphocytes.

¶ Animals with ATx 2 weeks before irradiation and marrow reconstitution.

### DISCUSSION

The present study clearly showed that adult thymectomy decreased the primary cytotoxic response of spleen cells against syngeneic tumour cells. This was not due to a decrease in general immunological competence of these spleen cells, because their cytotoxic response to allogeneic cells and minor histocompatibility antigens, and their antibody response to SRBC were not affected by ATx. Since this effect was not seen in 2 weeks after ATx, but became apparent 6–8 weeks later, it seems likely that a population of short-lived T lymphocytes which migrate from the thymus to the spleen is involved. Studies of T lymphocytes involved in graft-*vs*-host responses (Cantor and Asofsky, 1972) and cytotoxic responses to allogeneic cells (Wagner, 1973) have shown a heterogeneity within the T-lymphocyte population. One of the T-lymphocyte subsets (T1) bears a relatively high concentration of the Thy-1 antigen on the cell surface, is found in the thymus and spleen, and is reduced after ATx (Cantor *et al.*, 1975). The other subset (T2), bearing a lower concentration of Thy-1 antigen and being mainly found in lymph nodes, lymph and blood, is not affected by ATx (Cantor *et al.*, 1975).

More recently it has been shown that functionally distinct subsets of T lymphocytes differ in their Ly surface phenotype. In C57BL/6 mice, Ly-2,3 cells generate cytotoxic activity to allogeneic target cells (Cantor and Boyse, 1975; Huber *et al.*, 1976), while cytotoxic T lymphocytes for syngeneic tumour cells are Ly-1,2,3 (Shiku *et al.*, 1976). Moreover, it has been reported that ATx resulted in ~50% decrease in the proportion of Ly-1,2,3 cells, and a slight increase in the proportion of Ly-2,3 and Ly-1 cells (Cantor and Boyse, 1975). Although it has not been proved that the precursors of cytotoxic cells for syngeneic tumours are also Ly-1,2,3, in the present study anti-tumour cytotoxicity was generated *in vitro* using non-immune spleen cells, and the results suggest that the precursors may well be Ly-2,3, and support the possibility that precursors to cytotoxic cells capable of killing syngeneic tumour cells may arise from a subpopulation of T lymphocytes distinct from those capable of generating help or killing allogeneic cells.

The Ly phenotype of suppressor cells, specific for syngeneic tumour cells, is not known, whereas they have been shown to bear the Thy-1 antigen (Takei *et al.*, 1976; Fujimoto *et al.*, 1976*a*). These suppressor cells appeared first in the thymuses of tumour-bearing animals. However, our data show that in ATx tumour-bearing animals suppressor cells are present in lymph nodes at levels comparable to those found in intact animals. This indicates that the thymus is not required for the generation of these cells, and that its absence does not affect their formation in any way. ATx in this system appeared to have very little effect on tumour growth and survival time in general.

These essentially negative data might also indicate that thymus-derived cells in fact play no role in tumour immunity, and that observations on suppressor or cytotoxic cells in various situations might be entirely fortuitous. In order to examine this possibility, animals were severely T-cell-depleted by ATx followed by lethal irradiation, and reconstitution with syngeneic marrow cells. Tumour growth in these animals as well as in appropriate controls was followed. It can be seen that in our hands severely thymocyte-deprived animals did less well than other experimental groups, and succumbed more rapidly to metastatic tumour. These observations imply that tumour immunity and temporary tumour containment, at least with this tumour line, is dependent on the presence of an intact immune system. It should be noted that these observations are in direct contrast to work reported by others (Falk, Nossal and Falk, 1977), who found that thymocyte-deprived rats were completely resistant to implantation of a mammary carinoma. These kinds of contradictory data emphasize the complexity of the relationship of the animal host to its tumour, and imply that no single model can be considered comprehensive.

F. Takei is a research fellow of the National Cancer Institute of Canada. The work was supported by the National Cancer Institute of Canada (Grant No. 65-6062 and 65-6048).

### REFERENCES

CANTOR, H. & ASOFSKY, R. (1972) Synergy among Lymphoid Cells Mediating the Graft-*versus*-Host Response. III. Evidence for Interaction between Two Types of Thymus-derived Cells. *J. exp. Med.*, **135**, 764.

CANTOR, H. & BOYSE, E. A. (1975) Functional Subclasses of T Lymphocytes Bearing Different Ly Antigens. I. The Generation of Functionally Distinct T-cell subclasses is a Differentiative Process Independent of Antigen. *J. exp. Med.*, **141**, 1376.

CANTOR, H., SIMPSON, E., SATO, V. L., FATHMAN, C. G. & HERZENBERG, L. A. (1975) Characterization of Subpopulations of T Lymphocytes. I. Separation and Functional Studies of Peripheral T-cells Binding Different Amounts of Fluorescent anti-Thy 1.2 (Theta) Antibody Using Fluorescence-activated Cell Sorter (FACS). Cell. *Immun.*, **15**, 180.

CARNAND, C., ILFIELD, D., PETRANYI, G. & KLEIN, E. (1975) The Role of Thymus on Autosensitization against Syngeneic Normal and Malignant Cells. *Eur. J. Immunol.*, **5**, 575.

FALK, R. E., NOSSAL, N. & FALK, J. E. (1977) Two-factor Suppression of Effective Anti-tumor Immunity. *Proc. Canad. Fed. biol. Sci.*, **20**, 86.

FUJIMOTO, S., GREENE, M. K. & SEHON, A. H. (1976*a*) Regulation of the Immune Response to Tumor Antigens. I. Immunosuppressor Cells in Tumor bearing Hosts. *J. Immun.*, **116**, 791.

FUJIMOTO, S., GREENE, M. I. & SEHON, A. H. (1976*b*) Regulation of the Immune Response to Tumor Antigens. II. The Nature of Immunosuppression Cells in Tumor bearing Hosts. *J. Immun.*, **116**, 800.

GERSHON, R. K. & KONDO, K. (1971) Infectious Immunological Tolerance. *Immunology*, **21**, 903.

HUBER, B., CANTOR, H., SHEN, F. W. & BOYSE, E. A. (1976) Independent Differentiative Pathways of Ly 1 and Ly 2,3 Subclasses of T-cells: Experimental Production of Mice Deprived of Selected T-cell Subclasses. *J. exp. Med.*, **144**, 1128.

JACOBS, D. M. & BYRD, W. (1975) Adult Thymectomy Results in Loss of T dependent Mitogen Response in Mouse Spleen Cells. *Nature*, **255**, 153.

KERBEL, R. S. & EIDINGER, D. (1972) Enhanced Immune Responsiveness to a Thymus-independent Antigen Early after Adult Thymectomy. Evidence for Short-lived Inhibitory Thymus derived Cells. *Eur. J. Immun.*, **2**, 114.

MCMASTER, R. & LEVY, J. G. (1975) Immunosuppression of Normal Lymphoid Cells by Serum from Mice Undergoing Chronic Graft-*versus*-Host Disease. *J. Immun.*, **115**, 1400.

MILLER, J. F. A. P. & MITCHELL, G. F. (1969) Thymus and Antigen-reactive Cells. *Transplant. Rev.*, **1**, 3.

NACHTIGAL, D., ZAN-BAR, I. & FELDMAN, M. (1975) The Role of Specific Suppressor T Cells in Immune Tolerance. *Transplant Rev.*, **26**, 87.

PECK, A. B., ALTER, B. J. & LINDAHL, K. F. (1976) Specificity in T-cell mediated Lympholysis: Identical Genetic Control of Proliferative and Effector Phases of Allogeneic and Xenogeneic Reactions. *Transplant. Rev.*, **29**, 189.

SHIKU, H., TAKAHASHI, T., BEAN, M. A., OLD, L. J. & OETTGEN, H. F. (1976) Ly Phenotype of Cytotoxic T-cells for Syngeneic Tumor. *J. exp. Med.*, **144**, 1116.

SIMPSON, E. & CANTOR, H. (1975) Regulation of the Immune Response by Subclasses of T Lymphocytes. II. The Effect of Adult Thymectomy upon

Humoral and Cellular Responses in Mice. *Eur. J. Immun.*, **5**, 337.

TAKEI, F., LEVY, J. G. & KILBURN, D. G. (1976) *In vitro* Induction of Cytotoxicity against Syngeneic Mastocytoma and its Suppression by Spleen and Thymus Cells from Tumor bearing Mice. *J. Immun.*, **116**, 288.

TAKEI, F., LEVY, J. G. & KILBURN, D. G. (1977) Characterization of Suppressor Cells in Mice bearing Syngeneic Mastocytoma. *J. Immun.*, **118**, 412.

WAGNER, H. (1973) Synergy during *in vitro* Cytotoxic Allograft Responses. I. Evidence for Cell Interaction between Thymocytes and Peripheral T-cells. *J. exp. Med.*, **138**, 1379.